# Comparison of Cardiac and Non-Cardiac Biomarkers for Risk Stratification in Elderly Patients with Non-Massive Pulmonary Embolism

**DOI:** 10.1371/journal.pone.0155973

**Published:** 2016-05-24

**Authors:** Nicolas Vuilleumier, Aurélien Simona, Marie Méan, Andreas Limacher, Pierre Lescuyer, Eric Gerstel, Henri Bounameaux, Drahomir Aujesky, Marc Righini

**Affiliations:** 1 Division of Laboratory Medicine, Department of Genetics and Laboratory Medicine, Geneva University Hospitals and Faculty of Medicine, Geneva, Switzerland; 2 Department of Internal Medicine, Rehabilitation and Geriatrics, Geneva University Hospitals and Faculty of Medicine, Geneva, Switzerland; 3 Division of General Internal Medicine, Bern University Hospital and University of Bern, Bern, Switzerland; 4 Service of Internal Medicine, Lausanne University Hospital, Lausanne, Switzerland; 5 CTU Bern, Department of Clinical Research and Institute of Social and Preventive Medicine (ISPM), University of Bern, Bern, Switzerland; 6 Clinique de la Colline, Geneva, Switzerland; 7 Division of Angiology and Haemostasis, Geneva University Hospitals and Faculty of Medicine, Geneva, Switzerland; Boston University, UNITED STATES

## Abstract

Biomarkers unrelated to myocardial necrosis, such as cystatin C, copeptin, and mid-regional pro-adrenomedullin (MR-proADM), showed promise for cardiovascular risk prediction. Knowing whether they are comparable to cardiac biomarkers such as high-sensitive cardiac-troponin T (hs-cTnT) or N-terminal pro-Brain natriuretic peptide (NT-proBNP) in elderly patients with acute non-massive pulmonary embolism (NMPE) remains elusive. This study aims at comparing the prognostic accuracy of cardiac and non-cardiac biomarkers in patients with NMPE aged ≥65 years over time. In the context of the SWITCO65+ cohort, we evaluated 227 elderly patients with an available blood sample taken within one day from diagnosis. The primary study endpoint was defined as PE-related mortality and the secondary endpoint as PE-related complications. The biomarkers’ predictive ability at 1, 3, 12 and 24 months was determined using C-statistics and Cox regression. For both study endpoints, C-statistics (95% confidence interval) were stable over time for all biomarkers, with the highest value for hs-cTnT, ranging between 0.84 (0.68–1.00) and 0.80 (0.70–0.90) for the primary endpoint, and between 0.74 (0.63–0.86) and 0.65 (0.57–0.73) for the secondary endpoint. For both study endpoints, cardiac biomarkers were found to be independently associated with risk, NT-proBNP displaying a negative predictive value of 100%. Among non-cardiac biomarkers, only copeptin and MR-proADM were independent predictors of PE-related mortality but they were not independent predictors of PE-related complications, and displayed lower negative predictive values. In elderly NMPE patients, cardiac biomarkers appear to be valuable prognostic to identify very low-risk individuals.

***Trial Registration*:** ClinicalTrials.gov NCT00973596

## Introduction

An important aspect of risk stratification in patients suffering from non-massive pulmonary embolism (PE) is the timely identification of very low risk patients who could safely be eligible for outpatient treatment. [1–5]. To achieve this goal, different prognostic algorithms have been developed and rely either on clinically based scores or on biomarkers [1, 6]. Among the latter, the most extensively studied biomarkers are cardiac biomarkers reflecting either cardiac myocyte stretch, such as B type natriuretic peptides (BNP), or necrosis, such as cardiac troponins (cTn) [6–10], which may provide independent and incremental value over clinical prediction rules in the elderly population [9]. Nevertheless, there is a growing body of evidence indicating that non-cardiac biomarkers, including markers of kidney failure (Cystatin C) or markers of endogenous stress, such as copeptin, the C-terminal fragment of arginin-vasopressin, and mid-regional proadrenomedullin (MR-proADM), could be of interest to identify individuals at risk of death with different acute diseases, including PE patients [11–15]. Nevertheless, how these non-cardiac biomarkers would perform in comparison to cardiac biomarkers has never been studied.

In this “head-to-head” comparison, we aimed to i) compare the prognostic accuracies of non-cardiac biomarkers (cystatin C, ultra-sensitive copeptin, and MR-proADM) versus cardiac biomarkers (high sensitive cTnT (hs-cTnT) and N-terminal proBNP (NT-proBNP)), and ii) determine the evolution of their respective prognostic accuracies over time in patients with non-massive PE aged 65 years or older.

## Methods

### Patient population and study design

This study is an ancillary study of the SWITCO65+ study, a prospective multicenter cohort dedicated to assess long-term prognosis of patients ≥65 years with proven deep vein thrombosis or PE in five university and four high-volume non-university hospitals in Switzerland [9, 16]. All patients provided written informed consent before enrolment and all local research ethics committees (“Comité d’Ethique des Départements de Médecine Interne et Médecine Communautaire et de Premier Recours”) approved the study and the consent procedure. The detailed study methods were previously published [17].

In total, the main study enrolled 695 patients with acute PE between September 2009 and March 2012. PE diagnosis was defined as a positive spiral CT or pulmonary angiography, a high probability on ventilation perfusion scintigraphy, or a proximal deep venous thrombosis (DVT) documented on compression ultrasonography or angiography [16]. Briefly, exclusion criteria included catheter-related thrombosis, insufficient German or French speaking ability, conditions incompatible with follow-up (i.e. terminal illness), an inability to provide informed consent (i.e severe dementia) and previous enrolment in the cohort.

For this ancillary study, patients with massive PE, defined by a systolic pressure ≤ 90 mmHg according to the European Society of Cardiology guidelines [1], were excluded (10 patients), as well as patients withdrawing consent within one day from inclusion or denying use of data (8 patients) and PE patients with no blood sample or for which blood samples were taken more than one day after diagnosis (450 patients), leaving 227 patients eligible for this analysis. Baseline demographic characteristics and clinical data were prospectively collected by medical records review performed by trained research nurses.

### Patients' follow-up

All patients completed follow-up at one, three, twelve, and twenty-four months after enrolment. Follow-up included one telephone interview and two surveillance face-to-face evaluations during the first year of study participation and then semi-annual contacts, alternating between face-to-face evaluations (clinic visits or home visits in house-bound patients) and telephone calls as well as periodic reviews of the patient’s hospital chart. During each visit/contact, study nurses interviewed patients to obtain information about the date, type, and circumstances (i.e., following a fall) of bleeding episodes and VTE recurrences, and assessed whether the patient had died. If a clinical event had occurred, this information was complemented by reviewing medical charts and interviewing patients’ primary care physicians and/or family members who provided written informed consent.

### Definition of endpoints

Two predetermined endpoints were used for this study. The primary endpoint consisted in PE-related mortality defined as all deaths definitely or possibly related to PE, as previously described [9, 16, 17]. The secondary composite endpoint consisted in PE-related complications defined by the following outcomes: PE-related death, recurrence of venous thromboembolic events (VTE) or major bleeding, as previously reported [9, 16, 17].

All study endpoints were adjudicated based on the consensus of three clinical experts who were blinded to patient characteristics and biochemical results. Diagnosis of VTE during follow-up was established according to the usual criteria: for deep venous thrombosis on the basis of abnormal results on ultrasonography; for pulmonary embolism on the basis of ventilation-perfusion lung scan showing a high-probability pattern, CT or angiography showing intraluminal defects, or confirmation of a new PE on autopsy [16].

### Sample Collection

Venous blood samples were collected at the respective emergency departments of recruiting centres, within one day of the diagnosis. Samples were immediately centrifuged, frozen and stored at– 80°C until analyses. Details regarding blood sampling/processing were described elsewhere [18].

### Biochemical Analyses

All samples were sent for analyses to the Geneva University Hospitals in order to avoid bias related to analytical differences. Plasma concentrations of Cystatin C were measured on an Image^™^ immunonephelometer (Beckmann Coulter Brea, Ca, USA) using Dako Cystatin C reagents (Dako, Glostrup, Denmark). Copeptin and MR-proADM, were measured with a KRYPTOR^™^ system using immunoassays based upon time-resolved fluorescence technology (Thermo Scientific, BRAHMS AG, Hennigsdorf/Berlin, Germany). As no validated cut-offs exist for non-cardiac biomarkers in the context of risk stratification for PE, we used retrospectively determined cut-offs, as described in the statistical analysis section.

NT-proBNP and hs-cTnT were measured by electrochemiluminescence methods on Cobas e601 analysers (Elecsys^™^, Roche, Rotkreuz, Switzerland). For hs-cTnT, we used a cut-off of 14 ng/L corresponding to the value above the 99^th^ percentile of our healthy population with a coefficient of variation of 10% as recommended and endorsed by the third universal definition of myocardial infarction [19–20]. For NT-proBNP, we prospectively used the cut-off of 300 pg/ml validated for heart failure exclusion regardless of age and gender [21], which has also been reported to display negative predictive values above 98% for PE-related complications at different follow-up durations in all coming or elderly patients with non-massive PE [9–10].

### Statistical analysis

We estimated the cumulative incidence of outcomes for categories of biomarker levels using the Kaplan-Meier technique. For NT-proBNP and hs-cTNT, we used the prospectively defined and validated cut–offs of 300 pg/mL and 14 ng/L respectively [9–10]. As we aimed to focus on the negative predictive value of the biomarkers for both primary and secondary outcome, we arbitrarily set the cut-off for the non-cardiac biomarkers at the values corresponding to the 20^th^ percentile. We assessed associations between log-transformed biomarker values and the outcomes using Cox-regression. Results were reported as unadjusted and adjusted hazard ratios (HR) with corresponding 95% confidence intervals (CIs) and p-values. HRs were adjusted for the PESI score [2] concerning PE-related mortality and for the Geneva prognostic score (GPS) [22] for PE-related complications. For the calculation of the PESI and GPS, we assumed comorbidities and conditions as absent or normal if information was missing. The discriminative ability of the biomarkers for events at 1 month, 3 months, 12 months, and 24 months was assessed by the sensitivity, specificity, positive and negative predictive value, and Harrell’s C-statistic, which is equivalent to the area under the ROC curve (AUC) in the case of binary outcomes. All analyses were done using Stata 13 (Stata Corporation, College Station, Texas, USA).

## Results

### Patients’ baseline characteristics

Patients’ demographic characteristics and median biomarker values upon admission are listed in Table 1. Given the high exclusion rate (65%) due to the absence of blood sample or to blood sampling performed more than 1 day after diagnosis, we examined whether difference in patients’ baseline characteristic could be observed between analysed (n = 227) and non-analysed patients (n = 450). Body mass index and the proportion of female gender and patients with blood oxygen saturation below 90% were found to be significantly lower in the analysed patients group than in the non-analysed patients group. All other patient characteristics were comparable between the two groups (data not shown).

**Table 1:** Patients baseline characteristics.

Characteristic	% (n) or median (IQR)
	n = 227
Age, years	75 (69–81)
Female gender	41.4% (94)
DVT (any)	19.8% (45)
Proximal DVT	17.6% (40)
Systolic Blood pressure <100 mm Hg	1.8% (4)
Pulse rate ≥110 beats/min.	11.9% (27)
Respiratory rate ≥30 breaths/min.	4.0% (9)
Oxygen saturation <90%	9.7% (22)
Temperature <36°C	7.9% (18)
BMI (kg/m^2^)	27 (24–30)
Altered mental status	2.6% (6)
Diabetes mellitus	15.4% (35)
History of coronary heart disease	17.6% (40)
Heart failure†	10.1% (23)
Arterial hypertension	65.2% (148)
Cerebrovascular disease‡	8.8% (20)
Chronic lung disease¶	14.5% (33)
Chronic renal disease††	16.3% (37)
Smoker (current or past)	50.2% (114)
Estrogen therapy during the last 3 months	2.2% (5)
Immobilization during the last 3 months§	22.5% (51)
Major surgery during the last 3 months	14.5% (33)
History of VTE	30.0% (68)
History of DVT	17.6% (40)
Active cancer#	17.2% (39)
Cystatin C (mg/L)	0.99 (0.85–1.23)
Cystatin C > 20^th^ percentile (> 0.83 mg/L)	79.3% (180)
Copeptin (pmol/L)	11 (5–22)
Copeptin >20^th^ percentile (> 4.66 pmol/L)	79.7% (181)
MR-ProADM (nmol/L)	0.90 (0.68–1.19)
MR-ProADM >20^th^ percentile (> 0.66 nmol/L)	79.7% (181)
hs cTnT (ng/L)	17 (8–34)
hs cTnT >14 ng/L	57.3% (130)
NT-proBNP (pg/mL)	658 (229–2233)
NT-proBNP >300 pg/mL	68.7% (156)

Data were missing for respiratory rate (18%), oxygen (6%), temperature (2%), BMI (0.4%), smoking status (0.4%), and estrogen therapy (0.4%).

^†^Acute heart failure NYHA class II/IV during the last 3 months, known history of systolic or diastolic heart failure, left or right heart failure, forward or backward heart failure, or a known left ventricular ejection fraction of <40%.

^‡^History of ischemic or hemorrhagic stroke or a transient ischemic attack.

^¶^Chronic obstructive pulmonary disease, active asthma, lung fibrosis, cystic fibrosis, or bronchiectasies.

^††^Diabetic or hypertensive nephropathy, chronic glomerulonephritis, chronic interstitial nephritis, myeloma-related nephropathy, or cystic kidney disease.

^§^Fracture or cast of the lower extremity, bed rest >72 hours, or voyage in sitting position for >6 hours during the last 3 months

^#^Cancer (solid or hematologic) requiring surgery, chemotherapy, radiotherapy, or palliative care during the last 3 months.

Abbreviations:

DVT: deep venous thrombosis

BMI: body mass index

### Incidence of endpoint according to follow-up duration

The primary endpoint was met by 2.6% (6/227) of patients at 1 month, 4.0% (9/227) at 3 months, 5.3% (12/227) at 12 months and 5.7% (13/227) at 24 months. Almost 9% (20/227) of patients experienced PE-related complications at 1 month, 11.0% (25/227) at 3 months, 18.9% (43/227) at 12 months, and 24.2% (55/227) at 24 months. The number of events according to different follow-up durations is summarized in Table 2.

**Table 2:** Cumulative number and proportion of events according to follow-up duration.

	at 1 month % (n)	at 3 months* % (n)	at 12 months* % (n)	at 24 months* % (n)
**Primary Endpoint**				
PE-related mortality	2.6 (6)	4.0 (9)	5.3 (12)	5.7 (13)
**Secondary Endpoint**				
PE-related complications:	8.8 (20)	11.0 (25)	18.9 (43)	24.2 (55)
*PE-related mortality*	2.6 (6)	4.0 (9)	5.3 (12)	5.7 (13)
*VTE recurrence*	0.9 (2)	2.6 (6)	7.9 (18)	10.1 (23)
*Major bleeding*	6.2 (14)	7.0 (16)	10.6 (24)	13.7 (31)

* from baseline

### Non-cardiac and cardiac biomarkers values upon admission and their association with study endpoints

With the exception of cystatin C that showed no discrimination at any time during the follow-up, C-statistics reported below indicated that all other biomarkers displayed a significant discriminative power for the primary endpoint (Table 3).

**Table 3:** Evolution of sensitivity, specificity and predictive values over time.

PE-related mortality					
	C-statistics* (95% CI)	Specificity (95% CI)	Sensitivity (95% CI)	Positive predictive value (95% CI)	Negative predictive value (95% CI)
**1 month**					
NT-proBNP^1^	0.82 (0.66–0.97)	32.1 (26.3–38.5)	100.0 (61.0–100.0)	3.8 (1.8–8.1)	100.0 (94.9–100.0)
hs-cTnT^2^	0.84 (0.68–1.00)	43.4 (37.1–50.0)	83.3 (43.6–97.0)	3.8 (1.7–8.7)	99.0 (94.4–99.8)
Copeptin^3^	0.75 (0.52–0.98)	20.4 (15.6–26.2)	83.3 (43.6–97.0)	2.8 (1.2–6.3)	97.8 (88.7–99.6)
MR-proADM^3^	0.75 (0.49–1.00)	20.4 (15.6–26.2)	83.3 (43.6–97.0)	2.8 (1.2–6.3)	97.8 (88.7–99.6)
Cystatin C^3^	0.48 (0.16–0.81)	19.9 (15.2–25.7)	50.0 (18.8–81.2)	1.7 (0.6–4.8)	93.6 (82.8–97.8)
**3 months**					
NT-proBNP^1^	0.83 (0.70–0.95)	32.6 (26.7–39.0)	100.0 (70.1–100.0)	5.8 (3.1–10.6)	100.0 (94.9–100.0)
hs-cTnT^2^	0.83 (0.70–0.95)	44.0 (37.6–50.7)	88.9 (56.5–98.0)	6.2 (3.2–11.7)	99.0 (94.4–99.8)
Copeptin^3^	0.79 (0.60–0.97)	20.6 (15.8–26.5)	88.9 (56.5–98.0)	4.4 (2.3–8.5)	97.8 (88.7–99.6)
MR-proADM^3^	0.80 (0.60–1.00)	20.6 (15.8–26.5)	88.9 (56.5–98.0)	4.4 (2.3–8.5)	97.8 (88.7–99.6)
Cystatin C^3^	0.54 (0.28–0.79)	20.2 (15.4–26.0)	66.7 (35.4–87.9)	3.3 (1.5–7.1)	93.6 (82.8–97.8)
**12 months**					
NT-proBNP^1^	0.80 (0.69–0.91)	33.0 (27.1–39.6)	100.0 (75.7–100.0)	7.7 (4.5–13.0)	100.0 (94.9–100.0)
hs-cTnT^2^	0.81 (0.70–0.92)	44.2 (37.7–50.9)	83.3 (55.2–95.3)	7.7 (4.2–13.6)	97.9 (92.8–99.4)
Copeptin^3^	0.79 (0.66–0.92)	20.9 (16.0–26.9)	91.7 (64.6–98.5)	6.1 (3.4–10.6)	97.8 (88.7–99.6)
MR-proADM^3^	0.83 (0.68–0.97)	20.9 (16.0–26.9)	91.7 (64.6–98.5)	6.1 (3.4–10.6)	97.8 (88.7–99.6)
Cystatin C^3^	0.58 (0.39–0.78)	20.5 (15.6–26.4)	75.0 (46.8–91.1)	5.0 (2.7–9.2)	93.6 (82.8–97.8)
**24 months**					
NT-proBNP^1^	0.81 (0.71–0.91)	33.2 (27.2–39.7)	100.0 (77.2–100.0)	8.3 (4.9–13.7)	100.0 (94.9–100.0)
hs-cTnT^2^	0.80 (0.70–0.90)	44.4 (37.9–51.1)	84.6 (57.8–95.7)	8.5 (4.8–14.5)	97.9 (92.8–99.4)
Copeptin^3^	0.79 (0.67–0.92)	21.0 (16.1–27.0)	92.3 (66.7–98.6)	6.6 (3.8–11.2)	97.8 (88.7–99.6)
MR-proADM^3^	0.84 (0.70–0.97)	21.0 (16.1–27.0)	92.3 (66.7–98.6)	6.6 (3.8–11.2)	97.8 (88.7–99.6)
Cystatin C^3^	0.61 (0.42–0.79)	20.6 (15.7–26.5)	76.9 (49.7–91.8)	5.6 (3.0–9.9)	93.6 (82.8–97.8)

*For calculating the c-statistics, biomarker values were used continuous.

^1^ for values > 300 pg/ml

^2^ for values > 14 ng/L

^3^ for values > 20th percentile

### Risk prediction according to cardiac biomarkers

Regarding the primary endpoint, C-statistics for cardiac biomarkers ranged between 0.80 and 0.84 and tended to remain stable during the 24-month observation period. More precisely, NT-proBNP had a C-statistic of 0.82 (95% CI: 0.66–0.97) and 0.81 (95% CI: 0.71–0.91) after 1 month and 24 months, respectively. hs-cTnT had a C-statistic of 0.84 (95% CI: 0.68–1.00) and 0.80 (95% CI: 0.70–0.90) at 1 and 24 months, respectively (Table 3).

Cox regression analyses indicated that NT-proBNP was associated with a two-fold increased risk of PE-related mortality (HR: 2.46 per log-unit increase, 95% CI: 1.26–4.80) that remained stable after adjustment for the PESI score (HR: 2.30, 95% CI: 1.16–4.53). This two-fold increase in risk appeared to be stable over time with HRs of 2.30 (95% CI: 1.49–3.55) and 2.15 (95% CI: 1.38 to 3.36) for unadjusted and adjusted analyses, respectively, after a follow-up of two years (Table 4).

**Table 4:** Association of biomarkers with outcomes.

PE-related mortality				
	Crude HR (95% CI)	p-value	Adjusted HR* (95% CI)	p-value
**1 month**				
NT-proBNP^1^	2.46 (1.26 to 4.80)	0.008	2.30 (1.16 to 4.53)	0.02
hs-cTnT^2^	1.59 (1.17 to 2.16)	0.003	1.59 (1.16 to 2.16)	0.004
Copeptin^3^	2.39 (1.06 to 5.36)	0.04	2.15 (0.97 to 4.80)	0.06
MR-proADM^3^	3.54 (1.11 to 11.28)	0.03	3.13 (0.93 to 10.56)	0.07
Cystatin C^3^	0.67 (0.05 to 9.15)	0.76	0.43 (0.02 to 7.61)	0.56
**3 months**				
NT-proBNP^1^	2.64 (1.51 to 4.61)	<0.001	2.49 (1.41 to 4.41)	0.002
hs-cTnT^2^	1.56 (1.20 to 2.02)	<0.001	1.56 (1.19 to 2.05)	0.001
Copeptin^3^	2.87 (1.44 to 5.74)	0.003	2.62 (1.32 to 5.23)	0.006
MR-proADM^3^	4.51 (1.85 to 10.98)	<0.001	4.10 (1.63 to 10.29)	0.003
Cystatin C^3^	1.11 (0.16 to 7.75)	0.91	0.80 (0.10 to 6.75)	0.84
**12 months**				
NT-proBNP^1^	2.18 (1.40 to 3.40)	<0.001	2.04 (1.30 to 3.22)	0.002
hs-cTnT^2^	1.50 (1.18 to 1.90)	0.001	1.51 (1.17 to 1.93)	0.001
Copeptin^3^	2.99 (1.62 to 5.52)	<0.001	2.76 (1.50 to 5.07)	0.001
MR-proADM^3^	5.01 (2.36 to 10.64)	<0.001	4.60 (2.12 to 9.97)	<0.001
Cystatin C^3^	1.70 (0.37 to 7.69)	0.49	1.33 (0.26 to 6.85)	0.73
**24 months**				
NT-proBNP^1^	2.30 (1.49 to 3.55)	<0.001	2.15 (1.38 to 3.36)	<0.001
hs-cTnT^2^	1.48 (1.17 to 1.87)	0.001	1.49 (1.16 to 1.90)	0.002
Copeptin^3^	2.99 (1.65 to 5.40)	<0.001	2.76 (1.53 to 4.97)	<0.001
MR-proADM^3^	5.26 (2.57 to 10.75)	<0.001	4.84 (2.32 to 10.10)	<0.001
Cystatin C^3^	2.59 (0.74 to 9.05)	0.14	2.15 (0.57 to 8.19)	0.26

Biomarkers were log-transformed and used continuous. Effects (HRs) are expressed per one log-unit increase.

* adjusted for PESI

^1^ for values > 300 pg/ml

^2^ for values > 14 ng/L

^3^ for values > 20th percentile

At 30 days, hs-cTnT was also associated with increased risk of PE-related mortality (HR: 1.59 per log-unit increase, 95% CI: 1.17–2.16), which remained unchanged after PESI-adjustment (HR: 1.59, 95% CI: 1.16–2.16). Similar to NT-proBNP, the risk appeared to be stable during the whole follow-up period (HR of 1.48 (95% CI: 1.17–1.87) and 1.49 (95% CI: 1.16–1.90) for unadjusted and adjusted analyses, respectively, after two years). Cardiac biomarkers were independently associated with PE-related mortality at any time.

**Fig 1:**
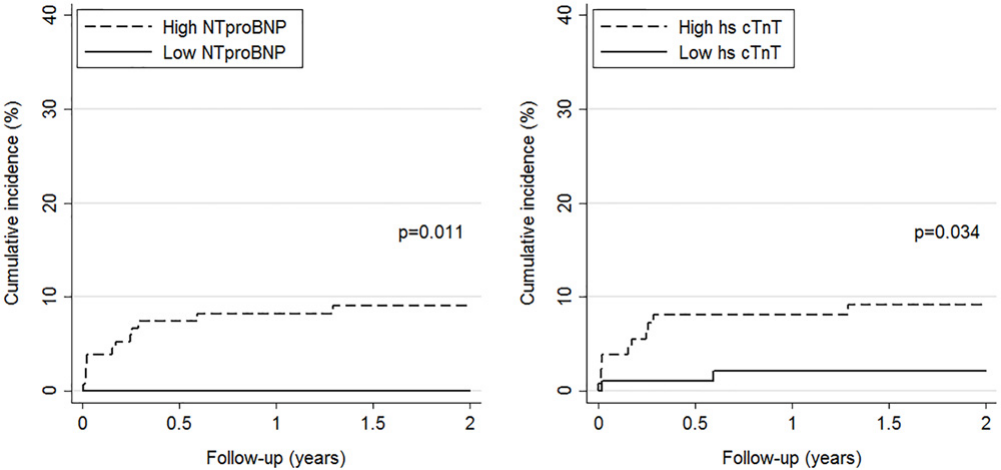
Cumulative incidence of PE-related mortality by level of NT-proBNP (left panel) and hs-cTnT (right panel). High versus low levels are based on pre-specified cut-offs (>300 pg/mL for NT-proBNP and >14 ng/L for hs-cTnT).

Kaplan-Meier curves showed that patients with NT-proBNP and hs-cTnT values above the pre-specified cut-off had a significantly higher cumulative incidence of PE-related death after 24 months than patients with values below the cut-off (9% vs 0% for NT-proBNP and 9% vs 2% for hs-cTnT, Fig 1).

Table 3 summarizes the evolution of cardiac biomarkers’ specificities, sensitivities and predictive values over time for the primary endpoint. At the pre-specified cut-off for NT-proBNP (300 pg/ml) and hs-cTnT (14 ng/L), negative predictive values remained over 100% (95% CI: 94.9–100) and 97.9% (95% CI: 92.8–99.4), respectively, for PE-related mortality during the whole follow-up. Table 3 also summarizes the evolution of non-cardiac biomarkers’ specificities, sensitivities and predictive values over time for PE-related death. Among non-cardiac biomarkers, copeptin and MR-proADM displayed highest negative predictive values, both reaching 97.8% (95% CI: 88.7–99.6) at 3, 12, and 24 months of follow-up.

Regarding the secondary endpoint, C-statistics for cardiac biomarkers ranged between 0.63 and 0.74 and were stable up to 3 months before decreasing gradually. For NT-proBNP, C-statistic evolved from 0.68 (95% CI: 0.57–0.78) after 1 month to 0.63 (95% CI: 0.56–0.70) after 24 months, while hs-cTnT values were 0.74 (95% CI: 0.63–0.86) after 1 month and 0.65 (95% CI: 0.57–0.73) after 24 months (Table 5).

**Table 5:** Evolution of sensitivity, specificity and predictive values over time.

PE-related complications					
	C-statistics* (95% CI)	Specificity (95% CI)	Sensitivity (95% CI)	Positive predictive value (95% CI)	Negative predictive value (95% CI)
**1 month**					
NT-proBNP^1^	0.68 (0.57–0.78)	33.8 (27.7–40.5)	95.0 (76.4–99.1)	12.2 (7.9–18.2)	98.6 (92.4–99.8)
hs-cTnT^2^	0.74 (0.63–0.86)	44.9 (38.3–51.7)	80.0 (58.4–91.9)	12.3 (7.7–19.1)	95.9 (89.9–98.4)
Copeptin^3^	0.60 (0.46–0.73)	20.8 (15.8–26.8)	85.0 (64.0–94.8)	9.4 (5.9–14.5)	93.5 (82.5–97.8)
MR-proADM^3^	0.60 (0.45–0.75)	20.3 (15.4–26.3)	80.0 (58.4–91.9)	8.8 (5.5–13.9)	91.3 (79.7–96.6)
Cystatin C^3^	0.47 (0.33–0.62)	19.3 (14.5–25.2)	65.0 (43.3–81.9)	7.2 (4.3–12.0)	85.1 (72.3–92.6)
**3 months**					
NT-proBNP^1^	0.68 (0.57–0.78)	34.2 (28.0–40.9)	92.0 (75.0–97.8)	14.7 (10.0–21.2)	97.2 (90.3–99.2)
hs-cTnT^2^	0.73 (0.62–0.84)	45.5 (38.8–52.4)	80.0 (60.9–91.1)	15.4 (10.2–22.6)	94.8 (88.5–97.8)
Copeptin^3^	0.64 (0.52–0.76)	21.3 (16.2–27.4)	88.0 (70.0–95.8)	12.2 (8.2–17.7)	93.5 (82.5–97.8)
MR-proADM^3^	0.62 (0.48–0.76)	20.3 (15.3–26.4)	80.0 (60.9–91.1)	11.0 (7.3–16.5)	89.1 (77.0–95.3)
Cystatin C^3^	0.50 (0.37–0.63)	19.8 (14.9–25.8)	72.0 (52.4–85.7)	10.0 (6.4–15.3)	85.1 (72.3–92.6)
**12 months**					
NT-proBNP^1^	0.65 (0.57–0.73)	35.9 (29.3–43.0)	88.4 (75.5–94.9)	24.4 (18.3–31.7)	93.0 (84.6–97.0)
hs-cTnT^2^	0.69 (0.60–0.77)	46.2 (39.1–53.4)	72.1 (57.3–83.3)	23.8 (17.3–31.9)	87.6 (79.6–92.8)
Copeptin^3^	0.60 (0.51–0.69)	21.2 (15.9–27.7)	83.7 (70.0–91.9)	19.9 (14.7–26.3)	84.8 (71.8–92.4)
MR-proADM^3^	0.59 (0.49–0.69)	20.7 (15.4–27.1)	81.4 (67.4–90.3)	19.3 (14.2–25.7)	82.6 (69.3–90.9)
Cystatin C^3^	0.49 (0.40–0.59)	18.5 (13.5–24.7)	69.8 (54.9–81.4)	16.7 (11.9–22.8)	72.3 (58.2–83.1)
**24 months**					
NT-proBNP^1^	0.63 (0.56–0.70)	35.5 (28.7–42.9)	81.8 (69.7–89.8)	28.8 (22.3–36.4)	85.9 (76.0–92.2)
hs-cTnT^2^	0.65 (0.57–0.73)	45.3 (38.1–52.8)	65.5 (52.3–76.6)	27.7 (20.7–35.9)	80.4 (71.4–87.1)
Copeptin^3^	0.58 (0.49–0.66)	19.8 (14.5–26.4)	78.2 (65.6–87.1)	23.8 (18.1–30.5)	73.9 (59.7–84.4)
MR-proADM^3^	0.57 (0.48–0.66)	20.3 (15.0–27.0)	80.0 (67.6–88.4)	24.3 (18.6–31.1)	76.1 (62.1–86.1)
Cystatin C^3^	0.49 (0.41–0.58)	18.6 (13.5–25.1)	72.7 (59.8–82.7)	22.2 (16.8–28.8)	68.1 (53.8–79.6)

*For calculating the c-statistics, biomarker values were used continuous.

^1^ for values > 300 pg/ml

^2^ for values > 14 ng/L

^3^ for values > 20th percentile

Cox regression analyses indicated that NT-proBNP and hs-cTnT were associated with an increased risk of 30-days complications by a factor of around 1.5 for a one log-unit increase (HR: 1.45, 95% CI: 1.07–1.96 for NT-proBNP, and 1.48, 95% CI: 1.22–1.80 for hs-cTnT), which remained stable after adjustment for GPS score (HR: 1.35, 95% CI: 0.99–1.85 for NT-proBNP and HR: 1.56, 95% CI: 1.26–1.92 for hs-cTnT). After two years, NT-proBNP and hs-cTnT levels were associated with a significant and similar risk of complications (HR: 1.31, 95% CI: 1.10–1.56 and 1.26, 95% CI: 1.08–1.46, respectively), which remained stable after GPS score adjustment (HR: 1.30, 95% CI: 1.08–1.56 for NT-proBNP and HR: 1.27, 95% CI: 1.09–1.48 for hs-cTnT). Except for NT-proBNP at 30 days of follow-up, cardiac biomarkers were independently associated with PE-related complications (Table 6).

**Table 6:** Association of biomarkers with outcomes.

PE-related complications				
	Crude HR (95% CI)	p-value	Adjusted HR* (95% CI)	p-value
**1 month**				
NT-proBNP^1^	1.45 (1.07 to 1.96)	0.02	1.35 (0.99 to 1.85)	0.06
hs-cTnT^2^	1.48 (1.22 to 1.80)	<0.001	1.56 (1.26 to 1.92)	<0.001
Copeptin^3^	1.43 (0.92 to 2.22)	0.12	1.30 (0.84 to 2.02)	0.23
MR-proADM^3^	1.84 (0.84 to 4.05)	0.13	1.50 (0.63 to 3.55)	0.36
Cystatin C^3^	0.57 (0.13 to 2.49)	0.46	0.40 (0.09 to 1.74)	0.22
**3 months**				
NT-proBNP^1^	1.55 (1.18 to 2.04)	0.002	1.47 (1.11 to 1.95)	0.007
hs-cTnT^2^	1.45 (1.22 to 1.73)	<0.001	1.52 (1.26 to 1.84)	<0.001
Copeptin^3^	1.73 (1.16 to 2.58)	0.007	1.60 (1.08 to 2.39)	0.02
MR-proADM^3^	2.35 (1.23 to 4.52)	0.01	2.08 (1.03 to 4.19)	0.04
Cystatin C^3^	0.94 (0.28 to 3.15)	0.92	0.68 (0.20 to 2.33)	0.54
**12 months**				
NT-proBNP^1^	1.40 (1.14 to 1.72)	0.001	1.38 (1.12 to 1.70)	0.003
hs-cTnT^2^	1.32 (1.13 to 1.54)	<0.001	1.35 (1.15 to 1.58)	<0.001
Copeptin^3^	1.38 (1.01 to 1.89)	0.04	1.35 (0.99 to 1.84)	0.06
MR-proADM^3^	1.71 (0.96 to 3.07)	0.07	1.61 (0.88 to 2.96)	0.13
Cystatin C^3^	0.81 (0.30 to 2.17)	0.68	0.68 (0.25 to 1.87)	0.46
**24 months**				
NT-proBNP^1^	1.31 (1.10 to 1.56)	0.003	1.30 (1.08 to 1.56)	0.005
hs-cTnT^2^	1.26 (1.08 to 1.46)	0.003	1.27 (1.09 to 1.48)	0.002
Copeptin^3^	1.28 (0.97 to 1.68)	0.09	1.26 (0.95 to 1.66)	0.11
MR-proADM^3^	1.48 (0.86 to 2.56)	0.16	1.42 (0.81 to 2.51)	0.22
Cystatin C^3^	0.87 (0.36 to 2.10)	0.76	0.78 (0.32 to 1.91)	0.59

Biomarkers were log-transformed and used continuous. Effects (HRs) are expressed per one log-unit increase.

* adjusted for GPS

^1^ for values > 300 pg/ml

^2^ for values > 14 ng/L

^3^ for values > 20th percentile

**Fig 2:**
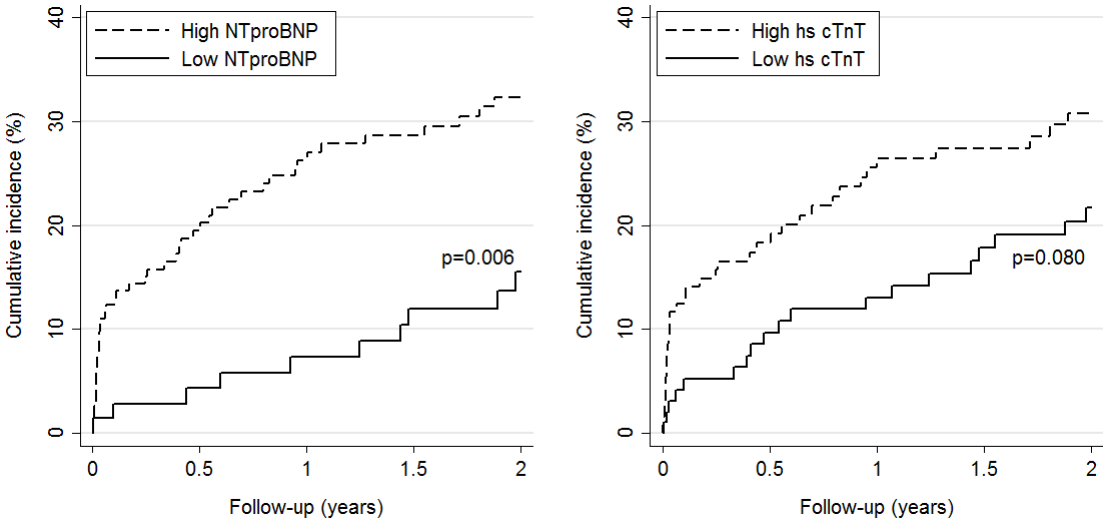
Cumulative incidence of PE-related complications by level of NT-proBNP (left panel) and hs-cTnT (right panel). High versus low levels are based on pre-specified cut-offs (>300 pg/mL for NT-proBNP and >14 ng/L for hs-cTnT).

For the secondary endpoint, Kaplan-Meier curves showed that only patients with NT-proBNP values above the pre-specified cut-off had a significantly higher cumulative incidence of event at 24 months than patients with values below the cut-off (32% vs 16%), whereas no significant difference was observed for hs-cTnT (31% vs 22%, Fig 2).

Table 5 summarizes the evolution of cardiac biomarkers’ specificities, sensitivities and predictive values over time for the secondary endpoint. At the pre-specified cut-offs for NT-proBNP (300 pg/ml) and hs-cTnT (14 ng/L), negative predictive values for PE-related complications were 98.6% (95% CI: 92.4–99.8) and 95.9% (95% CI: 89.9–98.4), respectively, up to 30 days and remained over 85.9% (95% CI: 76.0–92.2) and 80.4% (95% CI: 71.4–87.1) during the 2-year follow-up.

### Risk prediction according to non-cardiac biomarkers

Regarding the primary endpoint, non-cardiac biomarkers had lower C-statistic values than cardiac biomarkers. The C-statistic of non-cardiac biomarkers tended to increase over time. Furthermore, only copeptin showed a significant discriminative power at all times during the follow-up period, with C-statistics ranging from 0.75 (95%CI: 0.52–0.98) to 0.79 (95% CI: 0.67–0.92) after 1 and 24 months, respectively. MR-proADM did not show significant prognostic accuracy for PE-related mortality after one month (C-statistic: 0.75; 95% CI: 0.49–1.00), but was found to be a significant predictor for PE-related morality at 24 months (C-statistic: 0.84; 95%CI: 0.70–0.97). On the other hand, cystatin C levels did not show any predictive accuracy at any time of follow-up for PE-related mortality with C-statistics ranging between 0.48 (95% CI: 0.16 to 0.81) and 0.61 (95% CI: 0.42 to 0.79; Table 3).

Cox-regression analyses indicated that copeptin was associated with a two-fold increased risk of PE-related mortality at 1 month for each log-unit increase (HR: 2.39; 95%CI: 1.06–5.36), which was no longer significant after adjusting for the PESI score (HR: 2.15; 95%CI: 0.97–4.80). MR-proADM showed a four-fold increased risk for each log-unit increase (HR: 3.54, 95% CI: 1.11–11.28) after 30 days but, as with copeptin, did not remain significant after adjusting for the PESI score (HR: 3.13; 95%CI: 0.93–10.56). Interestingly, for copeptin and MR-proADM, hazard ratios tended to increase gradually over time and were found to be significantly associated with PE-related mortality after two years, independently of the PESI score (Table 4).

Differences in the cumulative incidence of PE-related mortality by level of non-cardiac biomarkers were not significant (Kaplan-Meier curves not shown).

Regarding the secondary endpoint, copeptin was the only non-cardiac biomarker that predicted PE-related complications at 3 months (C-statistic: 0.64; 95% CI: 0.52–0.76) and 12 months (C-statistic: 0.60; 95% CI: 0.51–0.69; Table 5). MR-proADM and cystatin C did not show significant discriminative ability at any time during the follow-up period. Moreover, none of the non-cardiac biomarkers (except for copeptin and MR-proADM at 3 months) displayed significant associations with the secondary endpoint in Cox regressions (Table 6) and Kaplan-Meier curve analyses (graphs not shown).

## Discussion

One of the major finding of this “head-to-head” comparison study is that cardiac biomarkers appear to have better discriminant power for PE-related mortality and PE-related complications than non-cardiac biomarkers. Hs-cTnT and NT-proBNP displayed adequate discriminant power with C-statistics exceeding the benchmark value commonly set at 0.80 for cardiac biomarkers [23]. Furthermore, for both study endpoints, NT-proBNP and hs-cTnT were in general independently associated with the endpoints when adjusted for the PESI and GPS scores. More interestingly, for PE-related mortality, a NT-proBNP value below 300 pg/ml had a negative predictive value of 100% up to 24 months of follow-up, and negative predictive values above 97% up to 3 months of follow-up for PE-related complications. In general, the negative predictive values for hs-cTnT were overall very similar to those obtained for NT-proBNP. In contrast, at the pre-specified cut-off values, both NT-proBNP and hs-cTnT displayed positive predictive values that were unsuitable, at least in the elderly population, to identify patients who could benefit from a more aggressive treatment in a rule-in strategy. These results are in line with the recent PEIHTO study indicating that a more aggressive management of normotensive PE patients based upon cardiac troponin elevation did not translate into clear clinical benefits [24].

Another significant contribution to the field resides in the observation that among non-cardiac biomarkers showing some promise for PE prognosis assessment [13–15], only MR-proADM, and copeptin appeared as valuable prognostic tools to predict PE-related mortality in patients with non-massive PE. Indeed, for this endpoint C-statistics indicated that both MR-proADM and copeptin had AUC values close to 0.80.

Cox regression analyses indicated that for PE-related mortality, the association was independent of the PESI score. The negative predictive values for PE-related mortality of these two biomarkers appeared to be slightly lower than the ones observed for cardiac biomarkers and we observed a substantial overlap of the 95% confidence intervals of the negative predictive values, which could be interpreted as equivalence between copeptin, MR-proADM and cardiac biomarkers. Nevertheless, the lower limits of the 95% CI for both copeptin and MR-proADM were lower than those of cardiac biomarkers for each time point and were below 88.7% for both study endpoints. Accepting a negative predictive value of 88.7% would mean that for rule-out purpose, one would accept the theoretical risk that up to 11.3% of patients deemed at low risk would in fact die of PE-related mortality within the 1^st^ month (and up to the third month of treatment), which would be unacceptably high for PE patients meant to be treated in an ambulatory setting. Along the same line of thoughts, MR-proADM and copeptin were at best found to be marginally predictive for PE-related complications according to C-statistics, and with the exception of copeptin and MR-proADM at 3 months, Cox regression analyses indicated that the association with PE-related complication did not remain significant for both these non-cardiac biomarkers after the adjustment for the GPS, which is the best validated prognostic determinant in PE patients for this composite endpoint [2].

The present results partly support the results of a smaller study indicating that MR-proADM was independently associated with increased mortality in PE patients, but do not lend weight to its superiority to NT-proBNP [15]. Nevertheless, consistent with previous findings in patients with other diseases indicating that MR-proADM could be a predictor of long-term mortality in heart failure patients [12], we observed that the prognostic accuracy of MR-proADM tended to increase over time, reaching C-statistics of 0.84 for PE-related mortality at 24 months, despite being a non-significant predictor at 1 month for this endpoint, or at any time point for PE related-complications. Due to the relative low numbers of events in this study, we cannot exclude a power issue explaining the absence of significant results for MR-proADM for short follow-up periods. Further studies are required to confirm the long-term prognostic value of this biomarker, as well as to define its impact on patient management. On the other hand, cystatin C had no discriminative properties for any of the study endpoints considered. The reason why we could not reproduce the promising results reported earlier for cystatin C [14] remains elusive. However, it could be related to the fact that only patients older than 65 years-old were considered in this study and that age is known to have a significant impact on MR-proADM levels [25] and on renal function.

The pathophysiological relationship between cardiac biomarkers and outcome in PE patients might be explained by the right cardiac ventricular strain induced by vascular obstruction caused by the occluding clot [1]. Similar mechanisms could explain the association between elevated levels of MR-proADM and outcomes in PE, as cardiomyocytes stretch and hypoxia have been shown to promote ADM gene expression [12–13]. For copeptin, which is supposed to be an integrative marker of endogenous stress related to any form of hemodynamic impairment [11], the possible physiopathological link relating elevated copeptin levels to PE outcome is less clear, but could certainly be viewed as a systemic response to impaired haemodynamics due to RV dysfunction, correlating therefore with PE severity.

This study has several limitations. First, the limited number of patients reaching the primary endpoint (n = 13) raises power issues, which could explain the absence of significant associations with outcomes mostly for MR-proADM and cystatin C. Therefore further larger studies are required to confirm the superiority of cardiac over non-cardiac biomarkers for risk stratification in non-high risk PE patients. Second, because the cut-offs for the non-cardiac biomarkers evaluated in this study have not been validated in PE patients, we used retrospectively defined cut-offs arbitrarily set at the 20^th^ and 80^th^ percentile. As a result, additional studies are required to define optimal copeptin cut-offs that could be used for PE risk stratification. Nevertheless, the results from the present study mostly indicate that using the previously validated cut-offs for hs-cTnT or NT-proBNP should be more adapted than using copeptin for risk stratification purposes. Finally, we cannot formally exclude that residual confounding could have biased the results of the present study. However, as the associations of cardiac biomarker with risk were found to be independent of PESI and GPS scores which take into account most of the well established prognostic determinants in PE, we consider this hypothesis as rather unlikely, at least for cardiac biomarkers. Finally, knowing whether the present results apply to PE patients younger than 65 years old remains to be demonstrated.

## Conclusions

Although current guidelines for risk stratification of PE patient recommend the use of clinical prediction rules, the present study indicates that in the elderly population, the use of cardiac biomarkers could represent an adequate alternative. Given their high negative predictive value reaching 100% for NT-proBNP for PE-related mortality and PE-related complications and the fact that they were shown to provide incremental prognostic value over clinical scores in the elderly population [14], cardiac biomarkers could represent valuable tools to identify low-risk patients with PE potentially eligible for outpatient treatment. Copeptin and MR-proADM appeared as valuable prognostic tool only for PE-related mortality prediction. On the other hand, cystatin C does not have prognostic accuracy. The safety of cardiac biomarkers at specific cut-off points to identify low-risk patients must be prospectively examined before their routine use can be adopted in routine clinical care.
